# Hematopoietic responses to SARS-CoV-2 infection

**DOI:** 10.1007/s00018-022-04220-6

**Published:** 2022-03-13

**Authors:** Shokrollah Elahi

**Affiliations:** Faculty of Medicine and Dentistry, School of Dentistry, Division of Foundational Sciences, Department of Oncology, and Li Ka Shing Institute of Virology, University of Alberta, 7020 Katz Group Centre, 11361-87th Ave NW, Edmonton, AB T6G 2E1 Canada

**Keywords:** Hematopoietic stem and progenitors, COVID-19, Stress hematopoiesis, Cytokine storm, Erythropoiesis

## Abstract

Under physiological conditions, hematopoietic stem and progenitor cells (HSPCs) in the bone marrow niches are responsible for the highly regulated and interconnected hematopoiesis process. At the same time, they must recognize potential threats and respond promptly to protect the host. A wide spectrum of microbial agents/products and the consequences of infection-induced mediators (e.g. cytokines, chemokines, and growth factors) can have prominent impact on HSPCs. While COVID-19 starts as a respiratory tract infection, it is considered a systemic disease which profoundly alters the hematopoietic system. Lymphopenia, neutrophilia, thrombocytopenia, and stress erythropoiesis are the hallmark of SARS-CoV-2 infection. Moreover, thrombocytopenia and blood hypercoagulability are common among COVID‐19 patients with severe disease. Notably, the invasion of erythroid precursors and progenitors by SARS-CoV-2 is a cardinal feature of COVID-19 disease which may in part explain the mechanism underlying hypoxia. These pieces of evidence support the notion of skewed steady-state hematopoiesis to stress hematopoiesis following SARS-CoV-2 infection. The functional consequences of these alterations depend on the magnitude of the effect, which launches a unique hematopoietic response that is associated with increased myeloid at the expense of decreased lymphoid cells. This article reviews some of the key pathways including the infectious and inflammatory processes that control hematopoiesis, followed by a comprehensive review that summarizes the latest evidence and discusses how SARS-CoV-2 infection impacts hematopoiesis.

## Introduction

SARS-CoV-2 infection exhibits a spectrum from asymptomatic or mild to moderate and severe form of the disease [1]. Unfortunately, a sub-population of these infected individuals becomes critically ill and develops acute respiratory distress syndrome (ARDS), a clinical phenomenon characterized by the development of bilateral lung infiltrates and hypoxemia [2], often accompanied by septic shock syndrome and organ failure [3, 4]. It has been demonstrated that SARS-CoV-2 infection is initiated by the viral surface spike glycoprotein (S protein) [5] adhering to the angiotensin-converting enzyme-2 (ACE2) for cell entry [6]. Subsequently, the S protein gets cleaved by the transmembrane protease serine 2 (TMPRSS2), the SARS-CoV-2 co-receptor, to establish the infection [6]. As a result, SARS-CoV-2 not only gains initial entry through ACE2 but also downregulates cell surface ACE2 expression such that this enzyme cannot mediate its protective physiological function in preventing acute lung failure [7]. Subsequently, downregulation of ACE2 in the respiratory tract is associated with enhanced neutrophil infiltration [8] that may result in angiotensin II accumulation and lung injury as reported in respiratory syncytial virus (RSV) and influenza H7N9 infections [9, 10]. The expression of ACE2 has been reported in a variety of cells, such as intestinal epithelial cells, renal tubules, endothelial cells, cerebral neurons, immune cells (e.g. alveolar monocytes/macrophages) [11], erythroid progenitors [12], and hematopoietic stem and progenitor cells (HSPCs) [13]. In addition to ACE2, SARS-CoV-2 may invade host cells via CD147 [14], a known red blood cell (RBC) receptor for *Plasmodium falciparum* [15]. CD147 is also expressed on mesenchymal stem cells and embryonic stem cells [16]. Another suggested receptor for SARS-CoV-2 is CD26 (DPP4) that is reported to interact with the SARS-CoV-2 spike protein [17]. CD26 was described as a functional receptor for the emerging human coronavirus-Erasmus Medical center (hCoV-EMC) that is widely expressed in both epithelium and immune cells except *B* cells [18]. Although it remains to be proven whether this protein could be a functional receptor for SARS-CoV-2, it is involved in stress hematopoiesis [19]. Recent studies have reported co-expression of ACE2, TMPRSS2, CD147 and CD26 on erythroid precursors/progenitors (CD71^+^ erythroid cells) [12]. Modeling studies have suggested that SARS-CoV-2 inhibits heme metabolism and induces hemoglobin denaturation [20], which influences the oxygen-carrying capacity of RBCs. Furthermore, the significant abundance of erythroid precursors and progenitors in the peripheral blood of COVID-19 patients has been reported to be associated with disease severity [12, 21], which is another supportive factor for the influence of SARS-CoV-2 infection on hematopoiesis. Mounting evidence has revealed the impact of SARS-CoV-2 infection on platelets as illustrated by thrombocytopenia and thrombosis [22]. Despite increased levels of granulocyte macrophage colony-stimulating factor (GM-CSF), declines in the proportion of monocytes, eosinophils and basophils have been reported in COVID-19 patients [23]. Similarly, reduced numbers of lymphocytes in the peripheral blood of COVID-19 patients have been observed, especially in those with more severe symptoms [4, 23, 24]. In contrast, COVID-19 disease is associated with substantial expansion of immature and dysfunctional neutrophils in the peripheral blood [25]. This is accompanied by a higher neutrophil recruitment to the lungs that has been linked to disease severity [23].

Moreover, dysregulated type I IFN expression, delayed antibody production, and cytokine storm are other indicators of the impact of SARS-CoV-2 infection on immune cells and hematopoiesis [26–29]. A recent study reported that more than 80% of the receptor-binding subdomain 1 of the spike protein of SARS-CoV-2 (RBD-SD1) strongly interacts with different cells from the bone marrow [30], implying the potential impact of SARS-CoV-2 infection on the bone marrow niche, in particular, HSPCs. In light of the above, SARS-CoV-2 infection affects the hematopoietic system. Therefore, this review initially provides a brief overview of steady-state versus stress hematopoiesis, and how hematopoietic cells respond to microbial pathogens to set the stage for discussing how SARS-CoV-2 infection impacts hematopoiesis.

## Hematopoiesis under physiological and stress conditions

Hematopoiesis is the process of blood cellular formation that continuously takes place in the bone marrow niches. This is a tightly regulated process to ensure the orchestrated proliferation of hematopoietic stem cells (HSCs) with self-renewal and differentiation capabilities to give rise to different lineage-committed hematopoietic progenitors [31, 32]. Subsequently, these various blood cell lineages upon maturation egress from the bone marrow into the blood circulation. HSCs are the only cells with the ability to differentiate and produce different blood cell lineages in the bone marrow niche throughout life [33]. The majority of generated blood cells are short-lived, except tissue-resident macrophages and memory B and *T* cells, ranging from a few days to months (e.g. granulocytes and RBCs, respectively). As a result, mature hematopoietic cells are required to be constantly renewed and replaced to ensure homeostatic peripheral blood cell supply. This is mediated through regulatory signals within the bone marrow niches from surrounding cells in the form of cell–cell interaction or soluble factors and/or from physical signals (e.g. temperature, low blood oxygen, contractile forces, and shear stress) [34–36]. Under the steady hematopoiesis conditions, the daily granulocyte production in an adult in estimated to be 0.5–1 × 10^11^ granulocytes [37]. Similarly, approximately 2 × 10^6^ new RBCs egress from the bone marrow per second and about the same number being cleared and recycled in an adult human individual [38]. However, the hematopoietic system has to rapidly and dramatically enhance its cellular output to meet the body’s increased demand when the steady condition is disturbed such as infections and compensates for the emergencies such as the loss of blood cells (e.g. injury and bleeding) [39]. Although the majority of HSCs are inactive (quiescent) during the steady homeostasis, they become activated to proliferate/differentiate in response to emergency hematopoiesis, such as naturally occurring events (e.g. infection and blood loss) or pathological insults (e.g. radiotherapy and chemotherapy) [39–41]. It is worth mentioning that high-dose radio–chemotherapy for hematological malignancies could result in damage to the bone marrow niche limiting proliferation and differentiation of HSCs [40, 42]. Despite several differences in the triggering factors, there are common underlying cellular and molecular mechanisms associated with naturally and pathologically occurring emergency hematopoiesis.

Among these, one of the major evolutionary factors influencing the hematopoietic system under stress conditions has been systemic bacterial infection [39, 43, 44]. The innate immune system is effectively capable of containing most bacterial infections at the insult site. However, dysregulated or insufficient innate immune response leads to systemic bacterial infection resulting in stress hematopoiesis characterized by leukocytosis, granulopoiesis, monopoiesis, the increased presence of neutrophil-precursors, and erythroid precursors [31]. While such a response to bacterial infections as a host defense mechanism to enhance myeloid output to fight off the infection is beneficial, the response of the bone marrow to a viral infection is somewhat different and more sophisticated. Viral infections can influence hematopoiesis and perturb bone marrow output under pathological conditions. In general, most acute viral infections may transiently alter hematopoiesis through the action of different mediators (e.g. type I IFNs, TNF-a, and lymphotoxin) as reported for influenza and lymphocytic choriomeningitis virus (LCMV) infections [45, 46]. However, chronic viral infections (e.g. human immunodeficiency virus (HIV) and hepatitis C virus (HCV)) have a profound and long-lasting impact on the functionality of the hematopoietic system [47, 48].

## Direct and indirect sensing of pathogens by HSCs induces stress hematopoiesis

**Fig. 1:**
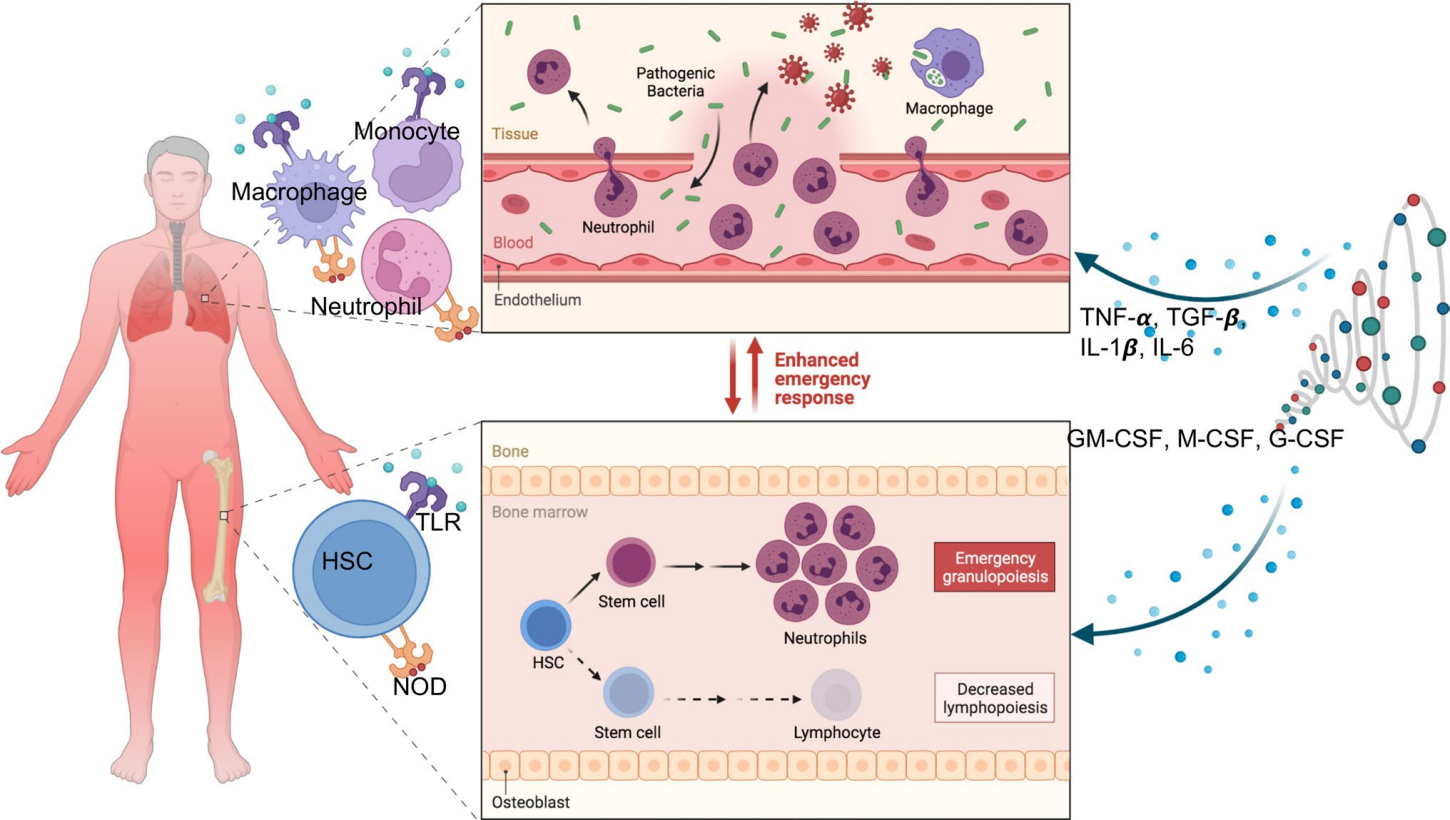
Model of stress hematopoiesis. Innate immune cells and endothelium via pathogen recognition receptors (PRRs) recognize pathogenic bacteria and/or viruses and subsequently become activated. This acute innate immune cell activation is associated with elevated levels of circulating cytokines (cytokine storm). Although these cytokines and other mediators are part of the innate immune response for the efficient clearance of pathogens, elevated and dysregulated cytokines are injurious to host cells. This complex interplay of cytokines with other immune cells may result in different outcomes. These cytokines can influence the functional properties of HSC and skew their differentiation towards myeloid but not lymphoid cells. Alternatively, HSC following recognition of PAMPs may become activated and differentiated to different stem cell progenitors depending on the nature of the pathogen/signal receiving

One may imagine many different scenarios result in stress hematopoiesis. First, in the context of infections, the naturally occurring steady-state hematopoiesis is shifted to infection-induced stress hematopoiesis upon sensing pathogen-associated signals. This normally takes place upon detection of different systemically disseminated viral, bacterial, fungal, or parasitic pathogens or their products. The systemic pathogens may signal directly to the HSCs (e.g. releasing pathogen-associated molecular patterns (PAMPs)) that bind to receptors contained in or on the HSCs (e.g. pattern recognition receptors (PRRs). Therefore, pathogen sensing is the crucial first step in the stress hematopoiesis cascade [49, 50]. The mechanistic details of pathogen detection during stress hematopoiesis in the bone marrow niche are complex. It requires the existence of specific cells capable of pathogen sensing, followed by the translation of pathogen sensing into enhanced hematopoietic stem and/or progenitor cells proliferation and differentiation (Fig. 1).

This scenario requires a) the availability of pathogen sensing cells in certain microenvironments with b) the probability of encountering a systemically spread pathogen, c) the ability to recognize the pathogen, and d) the capability of initiating the molecular signal of stress hematopoiesis upon such encounter [51].

In this scenario, this pathogen cell sensor such as innate immune cells would either require to be constantly patrolling different tissues for the identification of invading pathogens, or it would need to be widely present in different tissues for this purpose [51]. In contrast, another alternative mechanism would be the presence of certain cell type hidden in an otherwise sterile body compartment, and pathogen sensing by this cell type could be a strong indicator for a serious life-threatening microbial insult, providing a threshold for the distinction between a non-life-threatening and a serious systemic infection [31]. However, direct pathogen recognition by the presence of PAMPs that interact by receptors contained in or present on the HSCs is more acceptable. In agreement, PRRs in particular Toll-like receptors (TLRs) are well-defined sensors of evolutionarily highly conserved PAMPs [50, 52].

Nevertheless, TLR-expressing cells are widely abundant in tissues including both non-hematopoietic and hematopoietic origins. It is reported that HSPCs in mice and humans express TLRs and the nucleotide-binding oligomerization domain (NOD) like receptors (NLRs) [53–55] that constantly move out and re-entre the bone marrow [56]. Therefore, this highly increases the chances of pathogen encounter and recognition (Fig. 1).

By contrast, indirect sensing and initiation of stress hematopoiesis may occur via myeloid and non-hematopoietic cells. For example, it is well accepted that tissue-resident macrophages and mobile pathogen sensing phagocytes (e.g. monocytes) are highly equipped with different PRRs [57] and also capable of producing a wide range of pro-inflammatory cytokines and other immune modulators [58, 59]. In addition to hematopoietic cells, various non-hematopoietic cells, such as endothelial cells and mesenchymal stromal cells, express TLRs [60, 61], which enable them to sense pathogens and contribute to the inflammatory response in the course of stress hematopoiesis [60, 62]. Therefore, these shreds of evidence suggest that direct and indirect hematopoietic activation via microbial sensing play a crucial role in the regulation of stress hematopoiesis in the context of systemic infection and/or localized infection with systemic consequences.

## Cytokines, chemokines, and other soluble factors influencing stress hematopoiesis

Alternatively, alterations in the HSCs microenvironment by cytokines and other growth factors associated with infection may lead to stress hematopoiesis. Among these factors, so-called colony-stimulating factors including macrophage colony-stimulating factor (M-CSF) and granulocyte–macrophage colony-stimulating factor (GM-CSF) [57] can regulate hematopoiesis. Moreover, the role of IL-6 in the proliferation and differentiation of HSPCs following severe hemolytic anemia has been well studied [63]. IL-6 levels may regulate stress hematopoiesis in the bone marrow as demonstrated by an additive effect once examined in triple (G-CSF, GM-CSF, and IL-6) knockout mice [64]. Moreover, it is reported that MSC-derived IL-6 promotes the proliferation and differentiation of HSPCs in the bone marrow [65].

Interferons are inflammatory cytokines with well-documented properties in the context of different infections. However, their role in the context of hematopoiesis has not been free of controversy. Some reports suggest that IFN-α and IFN-γ directly activate quiescent HSPCs expressing both Types I and II IFN receptors [66, 67]; however, this concept was challenged by the observed anti-proliferative effects of IFN-γ on HSCs [68]. Besides, it is reported that CD8^+^ *T* cell-derived IFN-γ via release of IL-6 from bone marrow mesenchymal stem cells (MSCs) can promote HSPCs proliferation [65].

Also, the role of IL-3 as another cytokine with an important role in steady-state and stress hematopoiesis has been documented [69, 70]. However, the roles of IL-1α and IL-1β as classical inflammatory cytokines in stress hematopoiesis have not been fully elucidated. Recent evidence suggests that IL-1β promotes cell division and myeloid differentiation of HSCs [71] and is required for stress granulopoiesis [72]. Likewise, acute exposure of HSCs to IL-1β promotes their proliferation and instructs their differentiation towards myeloid fate [31]. Taken together, these observations support a role for cytokines and growth factors in stress hematopoiesis (Fig. 1).

## Viral infections and hematopoiesis

Both viral infections and the immune-mediated response to such infections can influence hematopoiesis. It has been documented that several viruses, such as HCV, human herpesvirus, and human cytomegalovirus, can infect HSPCs [48, 73–75], which results in the suppression of hematopoiesis. Likewise, it has been shown that HIV and simian immunodeficiency virus (SIV) infections are associated with the impairment of hematopoietic progenitors in humans and macaques, respectively [47]. Moreover, HIV and human T-lymphocyte virus type 1 (HTLV-1) by targeting microRNAs can manipulate key cellular pathways in hematopoietic cells resulting in malignancies [76, 77]. Overall, these results demonstrate that viral infections by directly targeting HSPCs may reduce the hematopoietic output. However, it is proposed that HSCs reside in the bone marrow niches to protect them from direct infection. Another possibility is that microbial products such as PAMPs can be circulated throughout the body as an indirect way to influence HSCs. Nevertheless, further studies are required to determine the susceptibility of HSPCs and their downstream progenitors to different viruses.

Moreover, as discussed in the infection sensing section, HSPCs via PRRs (e.g. TLRs) can recognize microbial pathogens. Notably, it is reported that human HSPCs express TLR-3, TLR7, TLR8 [54, 78, 79], which supports their direct ability of sensing viral PAMPs.

Finally, in response to viral PAMPs recognition by PRRs, non-immune and immune cells secrete a wide range of cytokines and chemokines. Viruses due to the differential nature of PAMPs/PRRs interactions, may induce divergent types of cytokines/chemokines [80]. Overall, pro-inflammatory cytokines, such as TNF-α, IL-6, type I IFN, and IL-1β, are common in viral infections [81]. For example, the chronicity of viral infections, such as HIV and HCV, results in dysregulation of different immune cells and the elevation of pro- and anti-inflammatory cytokines [82–85]. For instance, the elevation of IFN-α level is associated with the activation and proliferation of HSPCs in vivo [66].

According to another report, IFN-α transiently enhances HSPCs activation. However, chronic exposure to type I IFN returns HSPCs to dormancy status [86]. Apart from type I IFN, type II IFN also plays an important role in the impairment of HSPCs self-renewal capacity [68]. As mentioned above, recognition of viral PAMPs by PRRs can also result in the production of a variety of cytokines and chemokines (e.g. IL-6, TNF-α, IL-1β, TGF-β, GM-CSF, and M-CSF) as contributing factors to the proliferation and differentiation of HSPCs [87]. Taken together, HSPCs via direct detection of viral PAMPs can respond to viral infections. This results in the activation of signaling cascade, which may result in diverse biological outcomes from changes in lineage differentiation to cell death. Alternatively, pro- and anti-inflammatory cytokines and chemokines can influence the function/differentiation of HSPCs (Fig. 1).

## SARS-CoV-2 infection and hematopoietic progenitors

In general, HSPCs in the bone marrow give rise to different immune cell subpopulations in the periphery. Whether SARS-CoV-2 directly or indirectly influences the HSPC niche has been the subject of interest and debate. It has been reported that the RBD-SD1 probe interacted with bone marrow cells in an ACE2-independent manner. However, it was seen that SARS-CoV-2 upregulates the expression of ACE2 in human primary bone marrow cells [30]. The authors of this study have concluded that human bone marrow cells could be prone to SARS-CoV-2 infection. In support of this hypothesis, they have reported that different human stem cell lineages, such as CD34^+^CD133^+^lin^−^CD45^−^ cells, which may differentiate to HSCs and endothelial progenitor cells (EPCs), CD34^+^Lin^−^CD45^+^ HSCs and CD34^+^CD133^+^KDR^+^CD31^+^EPCs, express ACE2 and TMPRSS2 at the gene and protein levels [88]. In addition, it was observed that the viral spike protein activates NLrp3 inflammasomes in HSCs, which likely triggers their pyroptosis [88]. Post-mortem examination of COVID-19 patients has revealed evidence of direct viral infection of endothelial cells with accumulation of inflammatory cells. These findings suggest the susceptibility of ACE2 expressing endothelial cells to SARS-CoV-2 infection that facilitates endothelial cell injury in COVID-19 patients [89]. Similarly, expression of ACE2 on a great number of hematopoietic progenitor’s cells (HPCs) and up to 65% of HSCs derived from human cord blood has been documented [90], which is in agreement with another observation [91]. As such, someone can envision that direct infection of HSPCs can have a significant impact on HPSCs survival and renewal capacity in COVID-19 patients. Regardless of the infectivity of HSCs, HCSs/HPCs in response to SARS-CoV-2 viral spike protein undergo phenotypical and functional alterations [90]. These results may explain some of the observed changes in immune cell lineages in COVID-19 patients. Another study using single-cell RNA sequencing (scRNAseq) analysis has revealed the upregulation of genes associated with increased apoptosis and immune activation in human bone marrow mononuclear cells from COVID-19 patients [92]. In particular, dysregulated hematopoiesis characterized by a significant expansion of immature myeloid and marked decline of lymphoid progenitors was observed in those with a severe form of the disease [92]. Another group by studying transcriptional analysis of HSPCs from the peripheral blood of COVID-19 patients has revealed upregulation of genes associated with self-renewal and maintenance, such as CDK6, SOX4, and CHD4 [93]. These observations suggest that HSPCs in COVID-19 patients reshape in response to stress hematopoiesis. This was illustrated by a trend toward myeloid skewing, whereas frequencies of granulocyte/macrophage and common lymphoid progenitors were reduced in circulating HSPCs in COVID-19 patients with the severe and fatal disease [93].

## SARS-CoV-2 infection and lymphopenia

While COVID-19 starts as a respiratory infection, it has a significant impact on the hematopoietic system. One important feature of COVID-19 disease is lymphopenia [24, 94, 95]. Lymphopenia is a predictive factor for disease severity [95, 96] but appears to be reversed once patients recover [97]. Although lymphopenia has been reported to influence different lymphocyte subsets (e.g. *T* cell, *B* cell, and natural killer cell (NK cell)), some studies reported a more pronounced impact on the CD8^+^ *T* cell population [4, 98]. Concerning COVID-19 disease and *B* cells, one study has reported substantial inter-individual variation for *B* cell frequencies, ranging from extreme cytopenia or increased *B* cell count. Additionally, this study documented increased plasmablast (CD38^+^CD27^+^ cells) but reduced effector *B* cell frequencies in those with severe disease [99]. Consistent with most reports, we have observed a significant reduction in percentages of CD3^+^, CD4^+^, CD8^+^ T, and *B* cells in the peripheral blood of COVID-19 patients compared to healthy controls [100]. Notably, we found that lymphopenia was more CD8^+^ *T* cell biased especially in those in the intensive care unit (ICU) and the deceased had significantly greater lymphopenia compared to those who survived COVID-19 disease [100].

Transient lymphopenia (2–4 days) is commonly seen in different viral infections, such as human respiratory syncytial virus, human rhinovirus, influenza A H3N2 virus [101]. In contrast, lymphopenia in COVID-19 patients is more severe, persistent, and favorably impacts *T* cell subsets [98]. It is possible to speculate that peripheral lymphopenia in COVID-19 patients is due to the migration of lymphocytes to the site of active infection. However, autopsy studies of lung tissues and scRNAseq do not support this concept [102] and even a markedly lower abundance of CD8^+^ T cells has been observed in the upper respiratory tract of those with severe versus moderate disease [103].

The hallmark of SARS-CoV-2 pathogenesis is the induction of cytokine release syndrome. As such, cytokines directly or indirectly via other immune cells, such as neutrophils and dendritic cells (DCs), can induce lymphopenia [58, 104]. An accumulating body of literature illustrated the elevated concentration of plasma cytokines and chemokines in COVID-19 patients, in particular, in those in the ICU [28]. In agreement, we have reported the elevation of a wide spectrum of cytokines/chemokines including IL-6, TNF-α, IL-10, IL-12p70, IL-4, IL-15, GM-CSF, IL-17a, IL-8, IP-10, MCP-1, MIP-1α in the plasma of COVID-19 patients [27]. This cytokine storm is reported to be associated with the reduced number of lymphocytes. For example, a strong inverse correlation between IL-6 and IL-10 with total *T* cells and NK cells has been reported [105, 106].

Despite substantial reduction in CD4^+^ and CD8^+^ *T* cell counts, the proportion of hyper-activated *T* cells was elevated in COVID-19 patients [4]. Activated *T* cells following migration to the infection site (e.g. the lungs), although can eliminate virally infected cells, may promote *T* cell-dependent cytotoxicity [107]. Recent studies suggest that enhanced *T* cell-associated cytotoxicity could contribute to severe disease symptoms [108]. As such, the increased capability of IL-17 secreting *T* cells in critically ill patients has been proposed to heighten inflammation [100] and the subsequent activation and induction of neutrophils [109]. In line with these results, cells present in the lungs exhibited more IL-17 production capacity than those in the peripheral blood [110, 111]. These data further support a potentially detrimental role for IL-17 secreting cells in COVID-19 pathogenies. However, this concept merits further investigation.

Although the mechanism(s) underlying the lymphopenia in SARS-CoV-2 infection is not fully understood, the reduction in *T* cell count is a prominent feature of severe COVID-19 disease. Several potential mechanisms could be proposed to have direct or indirect effects on *T* cells. The use of different medications such as glucocorticoids because of their immunosuppressive properties on *T* cell proliferation [112] could also be a contributing factor as reported in SARS patients [113]. Enhanced apoptosis of *T* cells due to their hyper-immune activation status may in part explain their depletion [114]. Given the increased frequency of NKG2A^+^CD8^+^ *T* cells and the upregulation of co-inhibitory receptors (e.g. TIM-3 and PD-1), the concept of *T* cell exhaustion was proposed in COVID-19 patients [28, 115]. The heterodimeric receptor CD94/NK group 2-member A (NKG2A) is expressed by NK and CD8 *T* cells that induce an inhibiting signal [116]. However, overexpression of these co-inhibitory receptors can reflect *T* cell activation, as well. The concept of *T* cell activation has been widely described by the abundance of CD38^+^, HLA-DR^+^ and Ki67^+^ CD8^+^ *T* cells in COVID-19 patients [98, 117]. In agreement, we found a marked expansion in the frequency of activated *T* cells (expressing CD71, CD38, and HLA-DR) in COVID-19 patients. CD71, the transferrin receptor, not only is a marker of *T* cell activation but also a *T* cell proliferation marker (e.g. Ki67) [118]. Moreover, we observed a substantial increase in the frequency of PD-1, TIM-3, 2B4, VISTA, CD160, CD39, Galectin-9 (Gla-9), and TIGIT expressing *T* cells in COVID-19 patients [100]. Although *T* cell exhaustion is associated with the upregulation of co-inhibitory receptors [119–121], it is reported that the presence of co-inhibitory receptors was not associated with an impaired *T* cell but rather highly activated antigen-specific *T* cell phenotype in COVID-19 patients [100]. *T* cell exhaustion is characterized by functional impairment of *T* cells [119]. Therefore, overexpression of co-inhibitory receptors simply does not resemble an exhausted *T* cell phenotype [122]. More accurately, transient expression of co-inhibitory receptors on activated *T* cells is meant to prevent a massive and deleterious hyper-immune response. For example, the PD-1/PDL-1 axis mediates potent inhibitory signals to inhibit T effector cell function as a preventive mechanism of collateral damage management, without compromising antiviral immunity in an acute setting [123]. Alternatively, the hyperactivation signature of CD8^+^ *T* cells characterized by the upregulation of pro-apoptotic molecules, such as TRAIL, FAS (CD95), and/or caspase 3, may contribute to their elimination [99]. Hence, the clearance of highly activated CD4^+^ and CD8^+^ *T* cells might be a crucial mechanism associated with the maintenance of immune tolerance [124].

Another potential mechanism associated with the depletion of *T* cells, in particular, CD8^+^ *T* cells might be related to the elevated levels of plasma Gal-9 in COVID-19 patients[125] [27]. It has been reported that Gal-9 interaction with the Tim-3 receptor induces *T* cell apoptosis and regulates CD8^+^ *T* cell response in viral infections [126, 127]. In line with these observations, high levels of lymphocyte apoptosis have been observed in the spleen and lymph nodes of patients who expired of SARS-CoV-2 infection [128]. Therefore, it is possible to suggest that highly activated *T* cells in COVID-19 disease get eliminated by different mechanism (e.g. activation-induced cell death and Gal-9-mediated *T* cell apoptosis). Finally, the reduced plasma IL-7 concentration in COVID-19 patients may contribute to the observed lymphopenia in these patients [100]. IL-7 is an essential cytokine for *T* and *B* cell development and maintenance [129]. There is a possibility that stromal cells as a major source of IL-7[130], get impaired/damaged as a result of SARS-CoV-2-induced effects. This cytokine has a protective role in the setting of septic shock [129], which is frequently observed in severe cases of COVID-19 disease [3]. Indeed, IL-7 was recommended as a prime option to tackle chronic viral infection [131]. Impressively, IL-7 at 10 μg/kg was well-tolerated, resulting in increased lymphocyte count, and subsequently improved clinical outcomes in COVID-19 patients [132]. Therefore, lower plasma levels of IL-7 in COVID-19 patients imply another possible mechanism underlying lymphopenia in COVID-19 disease, which could be overcome by the therapeutic benefits of IL-7 therapy.

Viral load is another factor that may influence the frequency and functionality of the *T* cell response. Therefore, further investigations are required to understand the relationship between the viral load and *T* cell frequency/responses in COVID-19-infected individuals.

In addition to the impact of SARS-CoV-2 infection on B and *T* cells, peripheral NK cells are substantially depleted in COVID-19 patients. NK cells are innate lymphocytes that often divide into cytotoxic and cytokine-producing cells [84]. NK cells play an important role against viral infections and not only can directly target and eliminate virally infected cells but also can impact *T* cell response. The degree of NK cell activation can have either a protective role in limiting infection or a detrimental role by enhancing *T* cell-mediated immunopathology [133]. In general, human lungs are enriched in NK cells compared to the peripheral blood [133, 134]. Multiple reports indicated a lower frequency of peripheral blood NK cells in COVID-19 patients with moderate and severe disease [93, 106, 135]. A couple of studies based on single-sell landscape immune phenotyping in bronchopleural lavage fluid (BAL) of COVID-19 patients suggested that this reduced peripheral NK cell population may reflect redistribution to the site of infection [102, 103]. Overall, NK cells from COVID-19 patients exhibit an activated phenotype as determined by the upregulation of perforin, NKG2C and the killer-specific secretory protein of 37 kDa (Ksp37) [93, 136]. This was supported by the upregulation of genes associated with cytotoxic molecules, such as interferon-stimulated gene (ISGs) and proliferation markers [93]. At the protein level, NK cells from COVID-19 patients had elevated expression of activation markers (e.g. CD38 and CD69), cytotoxic proteins, such as perforin, and the death receptor ligand FasL [93, 136]. Moreover, NF-κB signaling was reported to be strongly upregulated in NK cells of COVID-19 patients [136], which is essential for transcription of genes encoding cytotoxic effector molecules, such as granzyme B and perforin, in NK cells [137]. An important observation by Maucourant et al. supported the expansion of armed NK cells (higher perforin content) in severe COVID-19 patients and similarly an increased abundance of adaptive NK cells in a subset of severe patients [136]. By contrast, another group reported evidence of NK cell exhaustion characterized by cytotoxicity defects in COVID-19 patients with a severe and fatal disease [93]. These observations imply that further investigations are needed to better understand the role of NK cells in host protection and immunopathology in the context of COVID-19 disease.

**Fig. 2:**
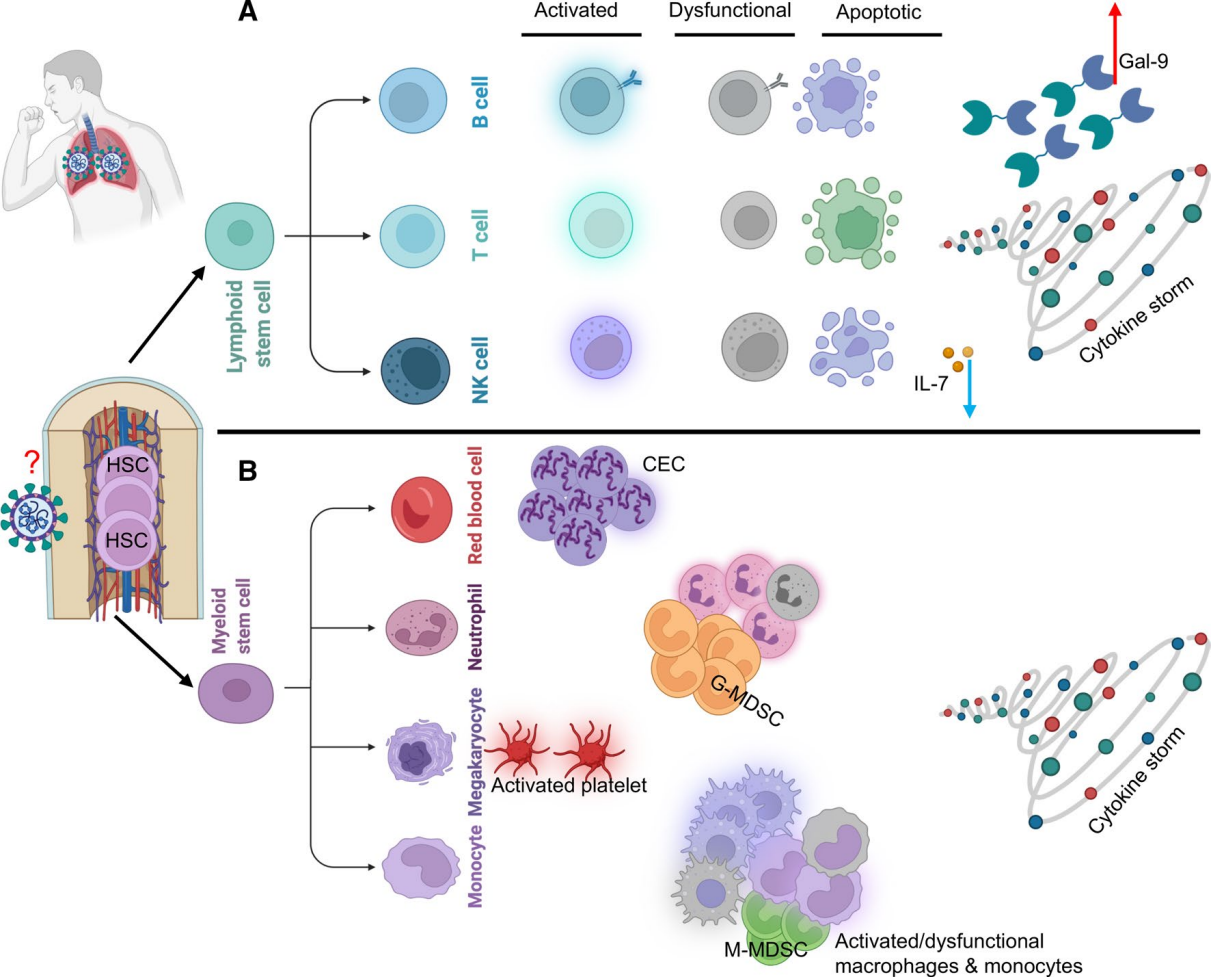
**A** This model illustrates the impact of COVID-19 disease on lymphoid lineage. Various studies have documented that COVID-19 disease is associated with T, B and NK cell activation. Also, some reports have shown dysfunctional/impaired lymphocytes. These highly activated lymphocytes become more prone to apoptosis as reported by the upregulation of apoptotic associated markers. Mechanistically this could be related to the direct impact of SARS-CoV-2 on HSC or associated with the general influence of the cytokine storm. Alternatively, a lower plasma IL-7 can impair lymphocyte proliferation but elevated levels of plasma Galectin (Gal-9) in COVID-19 patients may promote lymphocyte apoptosis. **B** In contrast, multiple reports indicated expansion of erythroid precursors/progenitors (CECs), increased number of mature and immature neutrophils (G-MDSC), activated platelets, expansion of M-MDSC, activated and/or dysfunctional macrophages and neutrophils in the blood of COVID-19 patients. In terms of mechanism(s), the direct effect of SARS-CoV-2 and cytokine storm on HSC have been proposed

Finally, the disease stage can have different effects on the fate and functionality of lymphocytes and could explain variability in different studies. For example, immune response characteristics are driven by virus-induced pathology in the early stage of infection but immune system-mediated immunopathology in the later stage of infection. Taken together, mounting pieces of evidence demonstrate lymphopenia as a predictive measure of disease severity in COVID-19 patients. Multiple mechanisms stated above and beyond could work together to lead to lymphopenia in COVID-19 disease (Fig. 2A). However, negligible levels of ACE2 expression on lymphocytes including *T* cells [12] do not support the concept of direct infection and elimination of lymphocytes by SARS-CoV-2[95]. However, further studies are required to examine this hypothesis.

## SARS-CoV-2 infection and myelopoiesis/neutrophilia

Neutrophils normally differentiate in the bone marrow and following maturation egress the bone marrow to become the most abundant leukocytes in the blood circulation [138]. Mature neutrophils have a short life cycle in the circulation and enter into the vessels or parenchyma of different organs but preferentially bone marrow, lungs, and spleen to perform their biological properties under hemostasis conditions [139]. This homeostatic migration of highly granule-armed neutrophils which depends on the chemokine receptor CXCR2 and Bmal1 protein [140], can cause collateral damage in vascularized tissues. As such, lungs are extremely prone to neutrophil-induced damage, which can be fatal as reported in animal models [141]. In humans, neutrophil-mediated vascular damage, pulmonary edema, and ARDS are observed in those with pneumococcal infections [142]. In a similar fashion, the influx of neutrophils into different tissues but especially lungs and kidneys, prime niches of SARS-CoV-2 replication, has been reported [143]. According to clinical data, neutrophilia is commonly observed in different cohorts of COVID-19 patients. The median neutrophil count is reported to be elevated in COVID-19 patients and it is higher in those with severe/critical type of disease [144, 145]. In line with this, neutrophilia is an indicator of disease severity as demonstrated in those with ARDS [145, 146]. Furthermore, among COVID-19 patients, it was found that non-survivors had higher neutrophil counts than survivors [145, 146]. Transcriptional studies have revealed that neutrophil phenotype is highly remodeled in COVID-19 patients, driven in part by upregulation of multiple alarmins (e.g. S100A8 and S100A9), CXCR1 (IL-8 receptor), and PADI4, which are essential for neutrophil extracellular trap activation and release (NETosis) [93]. Moreover, robust ISG signature in moderate and severe but not mild patients has been observed, which is likely because of sensing type I IFN at the site of infection [93]. It appears that neutrophils from non-survivors have a dominant ISG signature and PDL-1 expression phenotype [25, 93]. Overall, these observations suggest a more prominent activated neutrophil phenotype in severe and fatal COVID-19 patients [93].

Therefore, hyperactivated neutrophils are heavily armed by toxic compounds stored in their granules and the ability to form NETosis. In this scenario, neutrophils pose a serious threat to vascular tissues such as the lungs as shown in lung injury models and ARDS [141, 142]. These events are often seen following neutrophil activation and thrombosis, which can be exacerbated by NETs [147]. Therefore, these observations support neutrophilia as an indicator of disease severity. Local production of cytokines, such as GM-CSF, G-CSF, and M-CSF, plays an important role in the process of granulopoiesis of neutrophils and monocytes [148]. Analysis of blood samples from severe COVID-19 patients has revealed that neutrophils can be clustered in sequential stages, beginning with pro- and pre-neutrophils and mature neutrophils. Pre-neutrophils express primary neutrophil granule protein-encoding genes, pre-neutrophils constitutively express secondary- and tertiary granule-encoding genes, and mature neutrophils express canonical neutrophil-encoding genes [93, 149]. Further, gene expression analysis for these subpopulations has revealed high heterogeneity among low-density neutrophils (LDNs) [25]. LDNs are very scarce in the peripheral blood of healthy individuals but appear/expand under pathological circumstances, such as sepsis and stress hematopoiesis [150]. They exhibit immunosuppressive properties and are associated with dysfunctional immune responses [151]. It is reported that neutrophils from COVID-19 patients with mild disease were distinct from those with severe disease. Severe COVID-19 patients have more immature and pre- and pro-like neutrophils with signs of activation illustrated with the upregulation of CD64, RNAK, and RANKL but reduced expression of CD62L [25]. As compared to neutrophils from mild patients, severely ill patients exhibit increase in width of dispersion of activity and cell volume as indicators for increased immaturity and dysregulation. Moreover, neutrophils of severe COVID-19 patients display impaired oxidative burst upon stimulation, where their phagocytic activity is preserved [25]. Expanded LDNs or low-density granulocytes (LDGs) in COVID-19 patients exhibit four distinct subsets (promyelocytes, melocytes, bands and mature neutrophils) exemplified by the expression of CD33 [152], a granulocyte precursor marker. LDNs in COVID-19 disease are enriched with genes associated with immunosuppressive features [25] and functionality exhibit *T* cell proliferation in vitro [152]. Therefore, the emergence of LDGs can be attributed to granulocytic-derived suppressor cells (G-MDSCs) associated with *T* cell suppression. The immunosuppressive properties of G-MDSCs in cancer have been well documented [153] and these results confirm the expansion of similar cell phenotype in COVID-19 patients. Besides, the expansion of another subset of MDSCs defined as monocytic-derived suppressor cells (M-MDSCs) is reported in the peripheral but not lungs of COVID-19 patients with arginase-1-dependent immunosuppressive properties [154]. Likewise, G-MDSCs with immunosuppressive capabilities become highly abundant in the periphery of COVID-19 patients and their presence is associated with lymphopenia and disease severity [152, 155]. Mechanistically, G-MDSCs via iNOS and TGF-β inhibit *T* cell effector functions by inhibiting *T* cell activation, proliferation and differentiation [156, 157]. Overall, the increased proportion of G-MDSCs in the ICU admitted compared to non-ICU patients and non-survivors than survivors suggests a role for these cells in disease severity [156]. Due to the immunosuppressive properties of MDSCs, it is possible to speculate that MDSCs may exhibit a dual role. In the early stage of infection, their expansion may impair *T* cell response in clearing virally infected cells. In contrast, the host may benefit from the expansion of MDSCs in the later stage of the disease when immunosuppression may prevent lung injury/collateral damage. In addition to immature neutrophils, the mature neutrophil compartment is impacted by severe COVID-19 disease. This is illustrated by the segregation of mature neutrophils into non-activated, partially activated and highly activated subsets based on the loss of CD62L and the gain of CD64 and Ki67 [25]. Furthermore, it is reported that COVID-19 patients with severe disease have dysfunctional neutrophils characterized by impaired oxidative burst despite preserved phagocytosis activity [25]. These observations support the notion that SARS-CoV-2 infection results in substantial expansion and alteration in the neutrophil compartment (Fig. 2B). This is characterized by a combination of signs of inflammatory and anti-inflammatory neutrophils, which is similar to post-traumatic inflammatory conditions [158]. Last but not least, we have reported that neutrophils under steady condition express substantial levels of surface Gal-9 that is bound to CD44. Upon activation, neutrophils depalmitoylate CD44 and cause its movement out of the lipid raft [83, 159]. This process causes the shedding of Gal-9 from the surface of blood neutrophils and may in part explain the mechanism underlying elevated plasma Gal-9 in HIV and COVID-19 patients [160, 161]. Similarly, we have reported significantly lower levels of CD44 bound Gal-9 on neutrophils from COVID-19 patients, which imply the activation status of neutrophils in the context of COVID-19 disease [27].

In addition to changes in neutrophils, there are multiple reports regarding drastic changes within the myeloid department in COVID-19 patients especially in those with a severe form of the disease. For example, expansion of macrophages in the lungs of patients with the severe disease has been proposed to contribute to local inflammation and recruitment of other inflammatory cells, such as monocytes and neutrophils [102]. In line with this, enriched CCL2 and CCL7 chemokines in the BAL of COVID-19 patients are linked to the recruitment of CCR + monocytes [128]. Accordingly, scRNASeq analysis of BAL obtained from COVID-19 patients revealed an abundance of alveolar macrophages and monocytes in those with severe disease. Although patients with mild disease had an increased proportion of these cells compared to healthy individuals, this difference was more distinct in those with severe COVID-19 disease [102]. Of note, a sub-population of recruited macrophages into the lungs may promote fibrosis generation in COVID-19 patients [162], as reported in liver cirrhosis [163].

Moreover, a substantial increase in the proportion of blood inflammatory monocytes (CD14^+^CD16^+^ cells) capable of secreting IL-6 has been reported in COVID-19 patients with severe disease [164]. Similarly, a greater abundance of inflammatory monocytes (CD14^++^IL-1b^+^) enriched with inflammatory genes (e.g. CCL4, CXCR4, and ISGs) was found in COVID-19 patients [165]. These studies suggest that cytokine activation drives the emergence of dysregulated monocytes and the expansion of inflammatory monocytes to fuel inflammation in COVID-19 infection. A transcriptomic analysis recapitulated this phenotype shift by the upregulation of multiple ISGs and markers of immature and tolerogenic monocytes with disease severity [93, 166]. More importantly, monocytes from severe and fatal COVID-19 patients possess an MDSC-like phenotype capable of expressing *T* cells via depletion of L-arginine by the enzymatic activity of arginase I [25]. Also, monocytes from COVID-19 patients with severe disease downregulate HLA class II encoding genes and CD4 expression, which is associated with monocytes-to-macrophage differentiation and proinflammatory CD14 monocyte sub-population [93, 167]. These observations indicate that monocytes in severe and fatal COVID-19 patients expand and exhibit a heterogeneous population of highly activated to suppressive and dysfunctional phenotypes (Fig. 2B).

Interestingly, the monocyte-like MDSC does not express gene sets associated with monocyte progenitors and MDSCs but instead corresponds to mature monocytes [93, 167]. This implies that these dysfunctional and tolerogenic monocytes are not the products of stress myelopoiesis but instead, a phenotype acquired due to the inflammatory milieu of severe and fatal COVID-19 patients. In addition, the presence of SARS-CoV-2 nucleoprotein in ACE2 expressing tissue macrophages has been detected in post-mortem examination of tissues from COVID-19 patients [168]. It is unclear whether SARS-CoV-2 actively infected macrophages or the presence of viral protein reflects the uptake of virally infected cells by macrophages. However, it is possible that monocytes/macrophages actively get infected based on our observations that a substantial portion of these in the peripheral blood and the bone marrow cells expresses ACE2 and TMPRSS2 [12].

Despite many reports on the role of monocytes and macrophages in COVID-19 pathogenesis, several key questions remain to be addressed. First, it is still unclear whether direct viral sensing through PAMPs and PRRs is the mechanism of macrophage activation in COVID-19 patients as opposed to cytokine exposure. Second, the role of prior epigenetic remodeling in shaping monocyte responsiveness to SARS-CoV-2 is unknown. Third, the impact of microenvironment immune status (e.g. tissue, bone marrow, and blood) on monocyte/macrophage activation is another factor that should be taken into consideration. Finally, whether the tissue-resident versus monocyte-derived macrophage responds differently to SARS-CoV-2 remained to be determined.

## SARS-CoV-2 and erythropoiesis

RBCs are the most abundant cells in circulation and in healthy adults they are generated in the bone marrow. In a healthy human adult, ~ 2.5 × 10^11^ RBCs are generated and released from the bone marrow and the same number are recycled/cleared per day. RBCs are constantly produced under a highly orchestrated process regulated by multiple factors in adult bone marrow niches and local tissue microenvironments that control hematopoietic stem cell maintenance and survival [169]. Nonetheless, stresses, such as anemia, pregnancy, and chronic conditions, such as infection and cancer, may result in a process called extramedullary erythropoiesis, resulting in the generation of RBCs outside of the bone marrow (e.g. spleen and liver) [170, 171]. Moreover, acute hematopoiesis stresses may result in RBC morphological abnormalities, inability to respond to environmental cues, and premature egress from the bone marrow. In line with these facts, multiple reports have emerged to suggest that COVID-19 disease is associated with RBC abnormalities. For example, dysregulated iron homeostasis has been reported in SARS-CoV-2-infected individuals [172]. In particular, observational clinical studies have reported a strong association between anemia with significantly higher mortality, and functional iron deficiency with more severe inflammation and longer hospital stay in COVID-19 patients [172]. This study, also found that a ferritin/transferrin ratio of more than 10 was associated with a five-fold and an eight-fold increased risk of ICU admission and mechanical ventilation requirements, respectively [172]. Moreover, unusual RBC morphological abnormalities have been observed in COVID-19 patients. COVID-19 patients appear to have elevated RBC distribution width (RDW) [173] that is a standard component of RBC quality assessment. Although the specific mechanism(s) for the RDW alteration in COVID-19 disease remains to be determined, elevated RDW is associated with > 6 times increased mortality risk in these patients [173]. In general, elevated RDW is an independent risk factor associated with increased mortality from cardiovascular diseases, sepsis, pulmonary disease, viral hepatitis, stroke, anemia, and many other complications [174]. Although the reason behind RDW change in COVID-19 patients is not fully understood, it appears that this phenomenon in certain conditions delays the clearance of older RBCs and subsequently reduces RBC production. This may occur at the expense of increased leukocytes and platelets production, which occurs in inflammation [174, 175]. Moreover, several RBC structural abnormalities have been observed in blood smears of hospitalized COVID-19 patients [176]. The abundance of stromatocytes, knizocytes, and other membrane defects in COVID-19 patients [176], suggest that RBC injury and deformation may take place as a result of an immune-mediated response [177] or physical damage by the virus invasion [12]. In line with these observations, a comprehensive metabolomics, proteomics and lipidomics study of RBCs has revealed significant structural membrane alteration at the protein and lipid levels in COVID-19 patients [178]. In particular, alterations of the N-terminal cytosolic domain of band 3 (AE1) in RBCs from COVID-19 patients imply that these cells may not respond well to environmental cues in hemoglobin oxygen saturation/oxidant stress [178]. Thus, COVID-19 disease-mediated enhanced oxidation of structural proteins and impairment of membrane lipid homeostasis may change RBC deformability, likely contributing to the thromboembolic complications observed in COVID-19 patients with a severe form of the disease. In support of this hypothesis, a higher aggregation rate but lower deformability of RBCs in COVID-19 patients has been reported [179]. On the other hand, these alterations may be associated with changes in physical characteristics of RBCs, as the properties of plasma membrane interact with cytoskeleton to regulate their shape [180]. RBCs’ physical characteristics are critical for microcirculatory flow [181] and such changes may impair their optimal circulation resulting in hypoxia in COVID-19 patients. In agreement, deformability and heterogeneity of RBC deformation and size have been reported in COVID-19 patients [182]. Cell deformability is believed to be an essential marker for splenic clearance of RBC [183]. It is possible to speculate that severe deviation from normal deformability of RBCs results in their early removal by the spleen. This process may result in anemia and subsequently stress erythropoiesis. However, minor deviations from normal deformability may pass through the spleen unchecked. Due to a long RBC lifespan, such damaged cells may stay in circulation for months. Therefore, the abundance of such altered RBCs not only may influence oxygen supply but also put mechanical stress and influence the normal function of the spleen. Although further studies are required to better characterize the functional properties of RBCs in COVID-19-infected and -recovered individuals, it is likely to suggest that altered physical properties of RBCs because of their long lifespan may contribute to the long-term complications such as fatigue observed in COVID-19 patients (so-called “Long-COVID”)[184]. It is also predicted that the spike protein of SARS-CoV-2 interacts with the band 3 resulting in membrane damage and altered RBCs functions, such as reduced ATP release, decreasing vasodilation, and oxygen delivery in COVID-19 patients [185]. However, such predictive observations require rigorous biological validation. In addition, modeling studies have suggested that SARS-CoV-2 prevents heme metabolism and induces hemoglobin denaturation [20]. Therefore, hemoglobin alteration can compromise the oxygen-carrying capacity of RBCs in COVID-19 patients resulting in hypoxia. Interestingly, the main entry receptor of SARS-CoV-2, ACE2, has been reported in RBCs by proteomic analysis [186]. This implies that RBCs may get invaded by SARS-CoV-2. It is reported that RBCs can be directly infected (e.g. malaria) resulting in hemolysis [187]. In support of this concept and as we discussed above, several structural protein damages and changes in RBC membrane lipids have been reported in COVID-19 patients [178, 179, 182]. In addition to ACE2, SARS-CoV-2 may invade host cells via CD147 [188], a known RBC receptor for *P. falciparum* [15]. Lastly, CD26 is reported to interact with SARS-CoV-2 spike protein [17]. This protein is expressed on RBCs and is also involved in stress hematopoiesis [19]. In light of these pieces of evidence, it is possible that SARS-CoV-2 directly targets RBCs. Hence, damage of RBCs by SARS-CoV-2 subsequently results in their elimination or inability to perform oxygen delivery. This may result in hypoxia and stress erythropoiesis as a compensatory mechanism to meet the body’s oxygen supply. Stress erythropoiesis can result in the abundance of (CD71^+^ erythroid cells (CECs)) in the blood circulation [189]. CECs are expanded under physiological, such as the neonatal period, pregnancy [190–194], or pathological conditions, such as HIV infection, respiratory syncytial virus (RSV), and cancer [195–197]. Although the lack of nuclei cannot provide the cell machinery for viral replication in RBCs, the presence of nuclei in a subset of CECs (CD45^+^CD71^+^CD235a^+^ cells) enables them to support viral replication. In support of this hypothesis, it is reported that HIV-1 binds to CECs via CD235a and subsequently transfers the virus to uninfected CD4^+^ *T* cells. More importantly, CECs from HIV-infected individuals in the presence of antiretroviral therapy harbor infective viral particles and mediate the trans-infection of HIV-1 to CD4^+^ *T* cells [195]. Similar to the expansion of CECs in the circulation of HIV-infected individuals [195], we and others observed the same pattern in COVID-19 patients. However, the expansion of CECs in COVID-19 patients, in particular, those with severe disease is more drastic [12, 21, 198] (Fig. 2B). While CECs are almost undetectable or at very low frequency in the peripheral blood of healthy individuals [12, 199], they become expanded in the blood circulation of COVID-19 patients. As the disease progresses, the frequency of CECs increases from less than 1% in HCs to sometimes > 50% of total peripheral blood mononuclear cells (PBMCs) in those with severe disease. Also, a strong correlation between disease severity and proportion of these cells was observed [12]. Further studies confirmed the co-expression of ACE2 and TMPRSS2 by a sub-population of CECs defined as erythroid progenitors (CD45^+^CD71^+^CD235a^+^) [12, 21]. Moreover, these cells express CD147 and CD26, as other potential receptors for the virus. Notably, CD45 + CECs are the most dominant cells co-expressing ACE2 and TMPRSS2 in terms of proportion and intensity of expression followed by monocytes in the blood circulation of COVID-19 patients. It is worth mentioning that other immune cells, such as neutrophils, B, T, and NK cells, express a negligible amount of ACE2 and/or TMPRSS2 [12]. Subsequently, we found that CECs were highly susceptible to SARS-CoV-2 infection [12] and others have reported that the virus colonies were detectable in CECs up to two weeks after the initial infection [21]. Therefore, the infectivity of CECs to SARS-CoV-2 infection may provide a potential mechanism for the reported silent hypoxia in these patients. These pieces of evidence provide a cement foundation to the hypothesis that stress hematopoiesis in COVID-19 disease can expand CECs. In line with these findings, anemia has been considered as an independent risk factor related to severe COVID-19 disease [3, 200]. However, it is still unclear how CECs in the setting of COVID-19 disease get infected. It is possible to speculate that SARS-CoV-2 via damaged lung tissues enters the blood circulation to infect CECs. Under such circumstances, whether SARS-CoV-2 is capable of entering and invading the bone marrow niche where CECs are originated is unknown.

It is worth mentioning that a substantial proportion of human bone marrow resident CECs and monocytes co-expressing ACE2 and TMPRSS2 [12]. This suggests the permissibility of bone marrow resident cells to SARS-CoV-2 infection. Given the very high surface expression of ACE2 and TMPRSS2 on placental CECs and their susceptibility to SARS-CoV-2 infection [12], suggests the possibility of vertical viral transmission appears possible. This is further supported by the abundance of CECs in the peripheral blood of pregnant women at the later stage of pregnancy and placenta tissues [190, 201, 202]. This in part may support the reported viremia and placental transmission of SARS-CoV-2 [203].

**Fig. 3:**
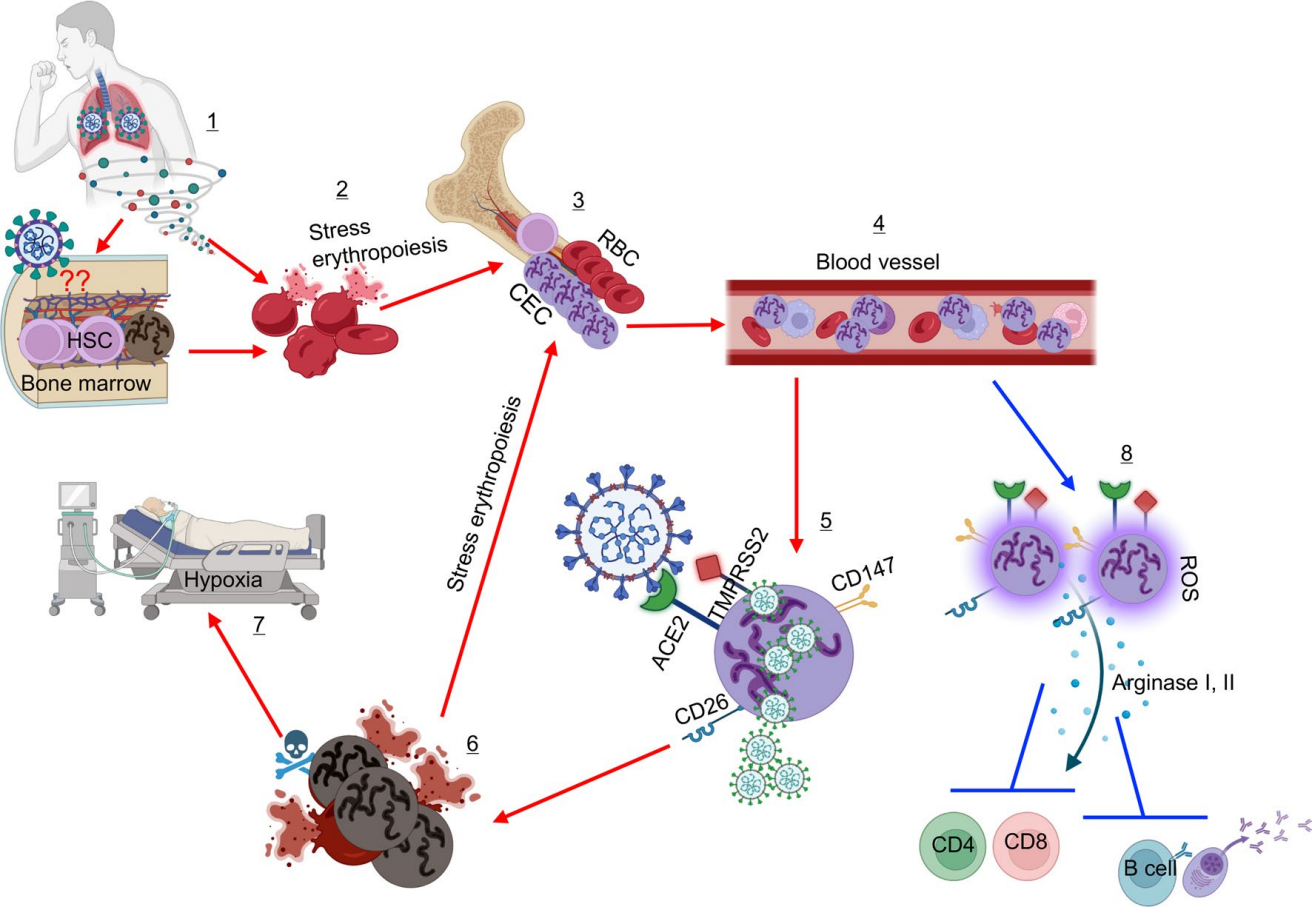
This model illustrates the impact of SARS-CoV-2 infection on hematopoiesis. Stress erythropoiesis characterized by the expansion of erythroid precursors/progenitors (CECs) in the peripheral blood of COVID-19 patients might be the result of direct invasion of HSCs in the bone marrow (1). In this scenario, erythroid progenitors get infected/lysed (2) and the bone marrow as a compensatory mechanism generates more CECs (3). Some of these CECs egress the bone marrow before maturing to RBCs and entering the blood circulation (4). This might be one potential reason for the massive number of CECs in the blood circulation of COVID-19 patients, especially in those with a severe disease. Alternatively, CECs may get exposed to the virus in damaged tissues of the lungs. It is reported that CECs are prone to infection (5) as they express the required receptors for SARS-CoV-2 (e.g. ACE2, TMPRSS2, CD147, and CD26). Therefore, infection of CECs results in their elimination (6), which results in a vicious cycle of stress erythropoiesis (3) and at the same time hypoxia in COVID-19 patients (7). In addition, expanded CECs via the secretion of arginase I, II, and ROS can suppress the proliferation and effector functions of *T* and *B* cells (8). Besides, there are multiple reports showing structural alterations/damages of mature RBCs in COVID-19 patients

Alternatively, the expansion of CECs can have another detrimental effect on the immune system of COVID-19 patients. In general, CECs exhibit immunosuppressive properties and suppress both innate and immune systems, as reported in different models [193, 204, 205]. CECs exhibit their immunosuppressive properties via cell–cell interactions (e.g. PD-1: PDL-1) or soluble factors, such as arginase I, arginase II, reactive oxygen species (ROS), and TGF-β[189, 194, 206]. In agreement with these reports, we found that CECs via the expression of arginase I, II, and ROS suppress *T* cell proliferation and cytokine production in vitro [12]. Moreover, we found an inverse correlation between the frequency of CECs with the percentages of *T* and *B* cells in the peripheral blood of COVID-19 patients [12]. These observations indicate that *T* and *B* cell effector functions are impaired by CECs in COVID-19 patients. Therefore, the invasion of CECs by the virus and acute inflammation results in stress hematopoiesis. This in turn enhances the production of CECs, which not only can be the target of SARS-CoV-2 but also impair the badly needed immunity against the virus. One may propose that such immunosuppression could be beneficial to COVID-19 patients since hyper-inflammation and cytokine storm are associated with disease severity [24]. As such, CECs may appear protective at the early stage of the disease to prevent a robust innate immune response [207]. Nevertheless, expansion of CECs in the peripheral blood of COVID-19 patients coincides with the disease progression, which is the time for the induction of an efficient adaptive immune response. Therefore, the absence of CECs at the early stage of disease deprives the host of their highly desired immunosuppressive properties but instead, their appearance later can compromise *T* cell effector functions and antibody production. The uncontrolled inflammatory response can itself damage the lungs via the excessive release of proteases, ROS, and pro-inflammatory cytokines [208]. Taken together, we predict a model in which SARS-CoV-2 directly or indirectly damages RBCs and through massive cytokine storm influences the bone marrow output. The bone marrow niche to compensate for this loss goes to stress erythropoiesis. As a result, immature RBCs (CECs) egress the bone marrow resulting in a massive abundance of these cells in the peripheral blood of COVID-19 patients. These cells then because of having high expression of ACE2 and TMPRSS2 might become the target of SARS-CoV-2. Viral replication in CECs will be associated with cell lysis and/or elimination by phagocytic cells. Alternatively, the bone marrow might be the target of the virus. The outcome of this vicious cycle will be decrease in circulating mature RBCs but instead increase in CECs and subsequently the commonly observed silence hypoxia in COVID-19 patients (Fig. 3).

## SARS-CoV-2 infection and thrombocytopenia

Platelets owing to their extremely high quantity (~ 1trillion in the blood of an adult human) and their properties to release immunomodulatory mediators are uniquely positioned to communicate with different immune cells. Platelets are enucleated with a short lifespan circulation (7–10 days) in humans after which they are cleared in the spleen and liver [209]. Thrombopoiesis mainly takes place in the bone marrow and is stems from the differentiation of HSCs into megakaryocytes. However, recent studies have highlighted the importance of the lung tissue as a platelet biogenesis niche, where megakaryocyte home and differentiate into platelets [210]. Multiple factors are involved in thrombopoiesis including thrombopoietin (TPO) that is produced mainly in the liver. Therefore, increased levels of thrombopoietin in inflammatory conditions (e.g. IL-6) can enhance thrombopoiesis [211]. Platelets express PRRs, such as TLRs, NOD-like receptors, glycoprotein, C-type-lectin-receptor family, and thus can sense/react to different microorganisms [212]. Thrombocytopenia in a common feature of viral (28%), bacterial (28%) and fungal (15%) infections [213]. During the SARS outbreak, about 20–55% of infected individuals presented thrombocytopenia that was considered as a mortality risk factor [113]. Similar observation with ~ 24–47% thrombocytopenia rate was reported in MERS patients [214]. Viral infection can contribute to thrombocytopenia by different mechanisms. Platelets can be activated by viral antigen–antibody complexes, direct detection/interaction with the virus, or host-associated inflammatory signals [215]. Platelet activation markers, such as P-selectin (a cell adhesion molecule) and platelet factor 4 (PF4), are highly increased in the plasma of COVID-19 patients, which supports the concept of enhanced platelet activation [216]. Platelet P-selectin is an important thrombo-inflammatory factor that regulates platelet–leukocyte interactions (e.g. neutrophils), recruits tissue factors and fibrin into platelet aggregates as a major step in thrombus generation [217]. Soluble P-selectin is reported to be elevated in ICU versus non-ICU patients and it is strongly associated with COVID-19-related mortality [218–220]. PFA, also called CXCL-4 is a chemokine stored in platelet granules that get released upon platelet activation [221]. In addition, transcriptional analysis of platelets has revealed the upregulation of genes related to platelet activation. In particular, upregulation of PPBP, CXCL7, and SELPLG was considered as biomarkers for intubation in COVID-19 patients [220]. Altogether, these data demonstrate that hyperactive platelets may contribute to hypercoagulability in COVID-19 disease that is associated with disease severity. Such activated platelets are easily removed from the blood circulation by the reticuloendothelial system [215]. Alternatively, viruses can directly influence or impair megakaryocytes to prevent platelet differentiation [222]. Platelets play an important role in recruiting leukocytes to endothelial surfaces, a crucial step in the procoagulant effect of viral infections as reported for SARS and MERS [223]. Activated platelets following interactions with macrophages, monocytes, endothelial cells, and lymphocytes play an essential role in the procoagulant process associated with viral infections [224].

Coagulopathy and thrombotic complications appeared to be a major aspect of COVID-19 pathogenesis as soon as the first cases were identified. COVID-19 disease is associated with a coagulopathy based on the elevation of procoagulant factors, such as fibrinogen and D-dimer, that have been linked to higher mortality [3, 225]. Coagulopathy was frequently seen in fatal cases of COVID-19 patients with prolonged prothrombin time [225]. A similar observation was made in another cohort where D-dimer was still associated with increased in-hospital mortality [226]. Increased D-dimer can reflect the disease severity, which is more elevated among critical cases of COVID-19 disease [3]. Identification of cases of thrombosis associated with antiphospholipid antibodies (e.g. anticardiolipin (aCL), anti-β2-glycoprotein I (aβ2GPI)2, and lupus anticoagulant (LA))[227, 228], resulted in the proposed preventive anticoagulation therapy in severe cases of COVID-19 infection by the International Society of Thrombosis and Hemostasis [229].

During disease progression, ~ 50% of COVID-19 patients appear to have elevated levels of D-dimer and all non-survivors possess a greater D-dimer. Likewise, disease severity has a positive correlation with D-dimer levels, suggesting increased thrombosis in these patients compared to those with mild disease [225]. Thrombocytopenia and increased D-dimer can be related to the hyperactivation of platelets and the coagulation cascade. In general, viral infections are associated with a systemic inflammatory responses that can tip the balance of anti/pro-coagulation homeostatic mechanisms [230]. Thrombocytopenia, platelet activation, and platelet interaction with other immune cells can have either protective or damaging consequences in the context of viral infections [231]. Although platelets are important in maintaining the basal barrier integrity of the alveolar capillaries, they can cause lung injuries under certain conditions [232, 233]. Platelets by aggregating leukocytes, enhancing endothelial permeability (e.g. IL-1β secretion), and directly interacting with endothelial cells play an important role in the pathogenesis of acute lung injury [234–236].

Furthermore, in contrast to viral pneumonia caused by other viruses, a greater pulmonary embolism incidence has been reported in COVID-19 patients [237]. There are several reports of blood vessels infection by SARS-CoV-2, which can result in vascular damage [89, 238]. As such the viral-induced endothelial lesion could be at the center of coagulopathy and hemostasis activation that promotes thrombotic disorders frequently seen in COVID-19 patients.

**Fig. 4:**
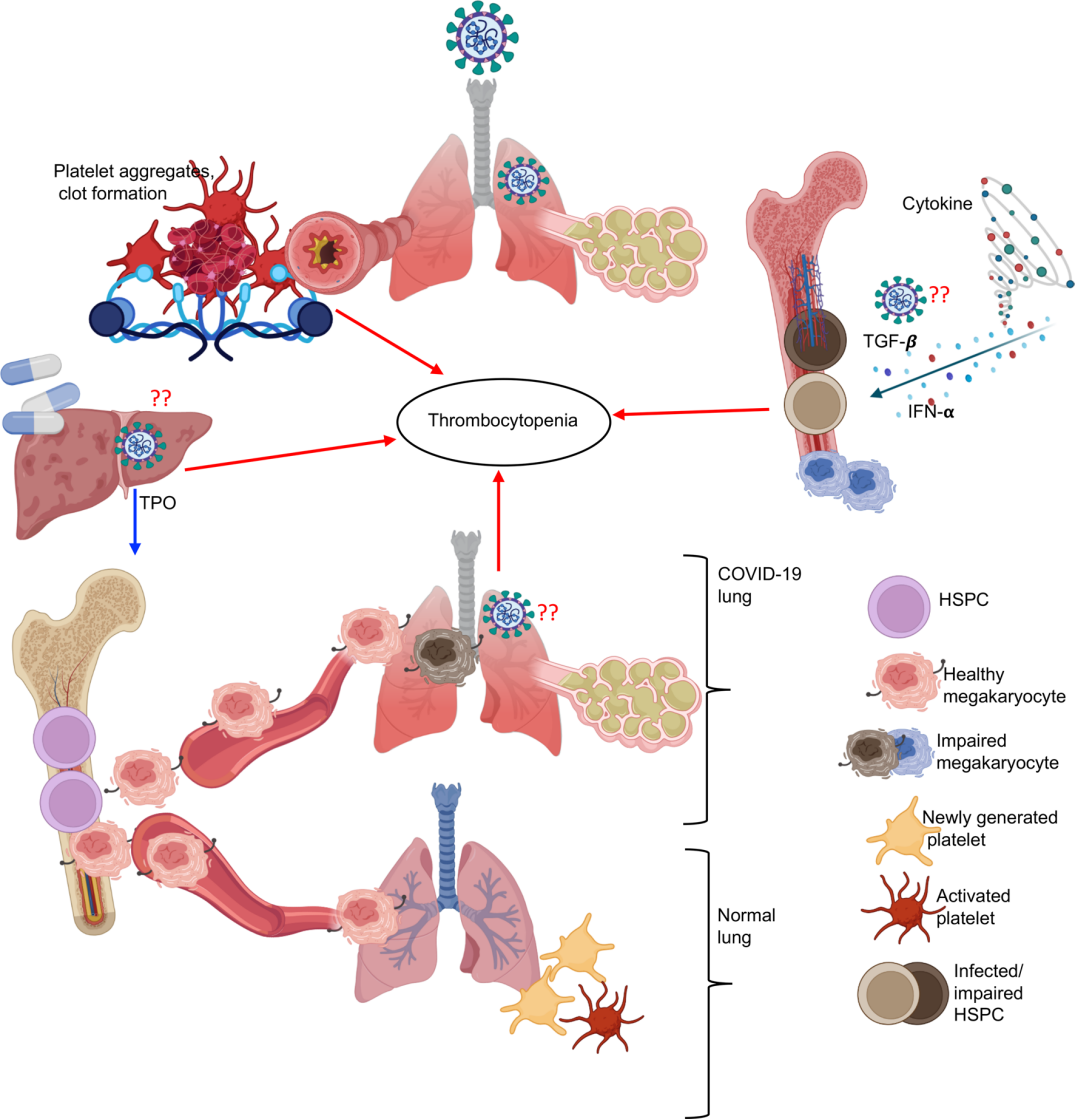
This model illustrates potential mechanisms associated with thrombocytopenia in COVID-19 patients. Coagulopathy is a major risk factor in severe COVID-19 patients. This is characterized by the formation of platelet aggregates resulting in platelets consumption at the site of infection. It has also been reported that plasma thrombopoietin (TPO) levels are declining in COVID-19 patients either because of the direct infection of liver cells by SARS-CoV-2 or drug toxicities as another potential factor in thrombocytopenia. Moreover, considering the reports of HSPCs susceptibility to the virus, it is possible to speculate that SARS-CoV-2 may impair HSPCs differentiation to megakaryocytes or make them dysfunctional. The cytokine storm in particularly elevated levels of plasma TGF-β and IFN-α can impair HSPCs differentiation into megakaryocytes. Another element that may imply the mechanism underlying thrombocytopenia in COVID-19 patients could be related to pathological damage to the lungs tissue. It is demonstrated that under steady physiological conditions, megakaryocytes are recruited to the lungs where they differentiate into platelets in mice. If this is the case for humans, then pathological alterations in the lungs following COVID-19 disease may impair/prevent this process

While mechanisms associated with thrombocytopenia in COVID-19 disease are not fully understood, several factors may contribute to this process that are illustrated in Fig. 4. This could be simply due to a high platelet consumption. SARS-CoV-2 replication triggers cell apoptosis, the release of different pro-inflammatory cytokines, and chemokines that can cause vascular injuries. This injury in the lung tissue including pulmonary endothelial cells leads to the activation, aggregation, and encapsulation of platelets at the infection site, which increases platelet consumption resulting in thrombocytopenia [213]. Another possible mechanism of thrombocytopenia could be related to the invasion of HSCs by SARS-CoV-2. Because of the expression of ACE2 and TMPRSS2 by HSCs and their susceptibility to SARS-CoV-2 infection [88, 90], it is possible to speculate that viral invasion and apoptosis of HSCs may suppress their differentiation into megakaryocyte progenitors. Moreover, cytokines and stem cell factors can influence the bone marrow niches thereby resulting in thrombocytopenia. For example, IFN-α inhibits megakaryocytes production in the context of SARS and MERS [239]. It also inhibits the expression of megakaryocytes’ transcription factor [240]. In addition to IFN-α, inhibition of megakaryocyte colony-forming units by increased levels of TGF-β in SARS infection has been reported [241]. These observations imply that cytokine storm, in particular, IFN-α and TGF-β may promote thrombocytopenia in COVID-19 patients. Moreover, SARS-CoV-2-infected patients with severe and fatal disease present massive lung damage characterized by diffuse alveolar damage, edema, pulmonary congestion, and pneumonia [24, 96].

It has been reported that the lungs are a reservoir for megakaryocytes and HPCs, and an important platelet biogenesis site accounting for 50% of total platelet production [210]. On the basis of this study that migrated megakaryocytes differentiate into platelets in the lungs, implying that thrombocytopenia might be the consequence of lung damage in COVID-19 patients. Lung damage, in turn, induces the activation of RAS and results in impaired functions of vascular endothelial cells and coagulation systems, which may further enhance platelet consumption and thrombocytopenia. Finally, the liver is the major source of TPO, and liver impairment/damage can impact the level of TPO production. Liver damage in COVID-19 patients might be related to the viral infection because the presence of the virus in liver tissue has been confirmed. Alternatively, it is possible to speculate that drugs hepatotoxicity and /or cytokine storm and pneumonia-associated hypoxia might contribute to the liver impairment and injury in severe COVID-19 patients [242]. Finally, it is speculated that autoantibodies against PFA elicit thrombus formation and thrombocytopenia by enhanced apoptosis and clearance of antibody-coated platelets in addition to platelet consumption in the coagulation process [243].

It is worth stating that thrombocytopenia is also associated with a larger mean platelet volume (MPV) in COVID-19 patients [244, 245]. This observation suggests that the enhanced production of immature platelets could be a response to reduced platelet numbers in COVID-19 patients. Reticulated or immature platelets have higher granule content, more residual mRNA, greater activation status, and mean volume compared with their older siblings [246, 247]. This could be another potential mechanism for the increased clotting events in COVID-19 patients since activated platelets are likely to promote platelet aggregations [248]. This concept is further supported by a positive correlation between immature platelet count with cardiovascular disease and mortality [246, 249]. Overall, thrombocytopenia and thrombotic complications are common in COVID-19 patients as mortality risk factors. Currently, available evidence implies that coagulopathy represents a combination of pulmonary platelet consumption, viral direct/indirect effects, and systemic elements.

## Concluding remarks and perspectives

As discussed above, the ability of the hematopoietic system to recognize and respond to threats, such as inflammatory, infectious, and injury stimuli, to regulate cellular output is a major evolutionary determinant of survival. Numerous studies over the past couple of years have demonstrated the profound impact of COVID-19 disease on the hematopoiesis system. Based on these findings, a complex picture is emerging, being composed of potential direct invasion of HSPCs and other progenitor cells by SARS-CoV-2. Alternatively, the excessive hyper-inflammation or cytokine release syndrome that is accountable for most of the lethality during severe COVID-19 disease might be linked to activation and enhanced proliferative capacity of the hematopoietic system. This may represent a state of stress and dysregulated hematopoiesis in COVID-19 patients with the severe and critical disease. Given the short life span of myeloid cells, except tissue resident macrophages, stress hematopoiesis enhances myeloid cell output to meet the body’s innate immune demand at the expense of reducing lymphoid output.

However, future research will need to comprehensively dissect the relative contribution of cytokine storm as well as direct effects of viral invasion in stress emergency hematopoiesis. Of note, hematopoietic aging through either extrinsic or intrinsic factors impairs hematopoiesis response. Given the overwhelming impact of COVID-19 disease on the elderly population, the potential impact of aging on hematopoiesis response in the context of COVID-19 is not fully understood which merits further investigation. Why is this of importance? A still unresolved issue is the question as to whether a mechanism is considered relevant in the process of stress hematopoiesis that differs in the elderly. Such knowledge may confer a survival advantage on the young. Although many studies included in this review helped to increase our understanding of hematopoiesis response to SARS-CoV-2 infection, many questions remain to be answered. I have mentioned some of these outstanding questions as follow: What are the molecular mechanisms by which COVID-19 disease impacts hematopoiesis? What are the possible long-term consequences of COVID-19 disease on the hematopoietic system? Is there any association between hematopoietic impairment and Long-COVID? What is the contribution of bone marrow niche versus tissue-resident HSCs to overall response to COVID-19 disease? What are special PAMPs of SARS-CoV-2 or danger-associated molecular patters (DAMPs) involved in stress hematopoiesis? What are the relative contribution of different cytokines and danger molecules such as Gal-9 in stress hematopoiesis?

## Data Availability

All the relevant data are included in the manuscript.
